# COVID-19 and VA Medical Education Training Programs: Influence on Trainees and Contributions of Trainees to VA’s Pandemic Response

**DOI:** 10.1007/s11606-026-10522-3

**Published:** 2026-05-27

**Authors:** Linda M. Kawentel, C. Ann Vitous, Sharon M. Nelson, Gezan M. Yahya, Joanna S. Doredla, Karen Sanders, Nicholas W. Bowersox

**Affiliations:** 1 VA QUERI Center for Evaluation and Implementation Resources, Ann Arbor, USA; 2 Capital College, School of Behavioral Sciences and Education, Pennsylvania State University at Harrisburg—Capital College, Middletown, PA, USA, Pennsylvania State University at Harrisburg—Capital College, Middletown, PA USA; 3 Department of Learning Health Sciences, University of Michigan, Ann Arbor, MI USA; 4 Department of Psychiatry, University of Michigan, Ann Arbor, MI USA; 5 VA Office of Academic Affiliations (OAA), Washington, D.C., USA; 6 Department of Surgery (Ophthalmology), Brown University, Providence, RI USA

**Keywords:** Graduate medical education, Crisis response, Pandemic response, Program evaluation

## Abstract

**Background:**

The US Department of Veterans Affairs (VA) has prioritized efforts to understand the influence of COVID-19 on VA clinicians and the overall VA system of care. Given the importance of health professions trainee (HPT) contributions to clinical and research activities, it is important to understand HPT contributions to VA’s work during the COVID-19 pandemic.

**Objective:**

This study evaluated the influence of the COVID-19 pandemic on VA HPTs and the contributions of HPTs to VA’s pandemic response.

**Design:**

Across selected VA sites, semi-structured interviews were used to gather perspectives from three participant groups: (1) HPTs across three disciplines at different levels of training, (2) service chiefs representing the disciplines in which the HPTs received training, and (3) educational leadership. Interviews were conducted in 2021.

**Participants:**

Stratified sampling was used to identify 10 VA medical sites, from which 83 participants were purposefully sampled.

**Approach:**

Descriptive content analysis was used to iteratively analyze collected data.

**Key Results:**

HPTs reported decreased learning opportunities and increased safety concerns related to COVID-19 during their training placements. HPTs were seen as providing important contributions to multiple aspects of VA’s efforts to respond to challenges related to COVID-19, including providing needed clinical care, supporting VA’s shift to telehealth, providing informational and emotional support to Veterans, helping with COVID planning and decision-making, and fostering a positive work environment.

**Conclusions:**

Although COVID-19 presented limitations to HPT involvement in VA activities, HPTs made meaningful contributions to care delivery and brought skills to support VA’s transition to virtual care delivery. HPTs are an important group to be included in future emergency response planning within VA.

**NIH Trial Registry Number:**

Not applicable.

**Supplementary Information:**

The online version contains supplementary material available at https://doi.org/10.1007/s11606-026-10522-3.

## INTRODUCTION

Health professions trainees (HPTs) are individuals appointed to temporary positions in Department of Veterans Affairs (VA) medical facilities who perform clinical or research training experiences to satisfy program or degree requirements^1^. As the largest training provider for HPTs within the USA through partnerships between 170 VA Medical Centers and 1800 academic institutions, VA trains more than 120,000 HPTs each year across multiple clinical disciplines^2^. VA has prioritized the education of HPTs and incorporated HPT placements into nearly every major facet of VA clinical and research efforts. Given the importance of HPT contributions to VA, it is important to understand the experiences of HPTs during the COVID-19 pandemic and how they supported VA’s response to COVID-19. This information may inform plans to involve HPTs in future VA emergency response efforts in ways that capitalize on their contributions and address their participation challenges.

Previous work highlights how COVID-19 affected training opportunities for clinical trainees outside of VA^3^. Relative to pre-pandemic periods and earlier stages of the pandemic, physician trainees across multiple clinical disciplines, including primary care/general practice^4,5^, surgery^6^, cardiology^7^, otolaryngology^8,9^, pain medicine^10^, and hematology/oncology^11,12^, experienced fewer training opportunities during COVID-19. As opportunities decreased, some trainees expressed disappointment that they were not able to offer meaningful contributions to their sites’ COVID-19 responses due to restrictive policies that limited patient interactions^13^, while others came together to create new policies that allowed them to mobilize and assist with their system’s virtual education and care coordination systems^14^. At the same time, more advanced physician trainees were involved in their sites’ COVID-19 response, being redeployed from their assigned work to assist with COVID-19 and non-COVID-19 care and filling in for attendings as appropriate and allowable^15^.

Within VA, previous studies have found lower levels of physician HPT training satisfaction and decreased likelihood of seeking future VA employment since COVID-19, partly due to decreases in patient volume or patient case mix^16^. However, a paucity of studies include the unique perspectives of trainees within the VA during the COVID-19 pandemic. In the study herein, pragmatic qualitative methods^17^ were used to evaluate the perspectives of undergraduate medical education (UGME, medical student) and graduate medical education (GME, physician resident and fellow) HPTs, service chiefs, and educational leaders within VA regarding the influence of the COVID-19 pandemic on VA HPTs and the contributions of HPTs to VA’s pandemic response, with the goal of improving overall learning at VA sites, future pandemic learning, and insight into what happened in the past during COVID-19. Such knowledge may help clarify the challenges that VA HPTs experienced during COVID-19, highlight contributions that HPTs made to VA’s COVID-19 response efforts, and point to opportunities to incorporate HPTs in planning for future emergency health crises.

## METHOD

### Study Design

This study is part of a larger evaluation using pragmatic qualitative methods, originally designed before the COVID-19 pandemic, exploring HPT contributions to the VA system of care. All HPT and supervisor interviews were conducted in 2021. During the development of the interview guide, OAA leadership expressed interest in understanding how the COVID-19 pandemic affected HPT training experiences and therefore one question specifically asking about the influence of COVID-19 on HPT training and contributions to VA’s COVID response was added to the interview. Interviewers also probed to understand how COVID impacted HPTs’ training experience. The findings presented in this report represent the results of these questions and investigated the experiences and contributions of UGME and GME HPTs within VA HPT programs during COVID across three medical disciplines: medicine, surgery, and psychiatry.

### Participant Sampling and Recruitment

A stratified sampling frame based on VHA Facility Complexity Model ratings^18^ was used to select VA medical centers, with targets varying by patient population, clinical services, educational and research missions, and administrative complexity. There was some geographic spread; however, most sites were concentrated in the Northeast and Midwest (Northeast 4; Midwest 4; South 1; West 1). Two interview sites from each VA complexity level were randomly selected. The Office of Academic Affiliations’ (OAA) Deputy Chief invited the Designated Education Officer (DEO) from each selected site to participate in the evaluation. The evaluation team sent follow-up emails detailing project information and the DEO’s desired role in participant recruitment. Seven of the ten selected sites agreed to participate: three sites that declined were replaced with the next randomly selected site from within their complexity level.

Purposeful sampling^19^was utilized to select participants from the interviewee groups of interest with DEOs assisting in recruitment. DEOs identified interview participants and facilitated outreach to selected groups. Participants were organized into three interview groups at each site: (1) HPTs at different training levels, (2) service chiefs representing the disciplines in which HPTs received training, and (3) educational leadership. For the HPT groups, the goal was to include one individual from each of the following levels: medical students, postgraduate year (PGY)-1 residents, PGY-3 residents, and physician sub-specialty fellows (the latter required for 1a complexity sites but optional for others). VA service chiefs were recruited from medicine, surgery, and psychiatry, with additional disciplines invited at the DEO’s recommendation. Site educational leader interview groups included site Chief of Staff and DEO. Due to scheduling issues, three participants were interviewed individually. A total of 30 groups were generated, ranging from one to six participants. Recruitment continued until sampling frame requirements were met^20^. In total, 83 participants took part in group interviews (Table 1). Demographic information was not collected, as it was not deemed relevant to the evaluation’s guiding questions by operational partners.

**Table 1:** Interview Group Characteristics (*N* = 33 Interviews)

Interview number	Interview group	Participant role*	Total participants
1C	Chief	Psychiatry 1, Psychiatry 2	2
1E	Executive Leader	COS, DEO	2
1 T	HPT	PGY-1, PGY-3	2
1T_r**	HPT	PGY-3	1
2C	Chief	Medicine, psychiatry	2
2E	Executive Leader	COS, DEO	2
2 T	HPT	Medical student, PGY-3	2
3C	Chief	Psychiatry, surgery	2
3E	Executive Leader	COS, DEO	2
3 T	HPT	Medical student, PGY-1, PGY-3, Chief Resident	4
4C	Chief	Medicine, psychiatry, surgery	3
4E	Executive Leader	COS, DEO	2
4T_s**	HPT	Medical student	1
5C	Chief	Medicine, psychiatry, surgery	3
5E	Executive Leader	COS, DEO	2
5 T	HPT	Medical student, PGY-1, PGY-3	3
5T_f**	HPT	Fellow	1
6C	Chief	Medicine, psychiatry, physical medicine rehab	3
6E	Executive Leader	COS, DEO	2
6 T	HPT	PGY-1, PGY-3	2
7C	Chief	Medicine, surgery	2
7E	Executive Leader	COS, DEO	2
7 T	HPT	Medical student, PGY-1, PGY-3, Chief Resident	4
8C	Chief	Medicine, psychiatry 1, psychiatry 2, psychiatry 3, psychiatry 4, surgery	6
8E	Executive Leader	COS, DEO	2
8 T	HPT	Medical student, PGY-1, PGY-3 (2)	4
9C	Chief	Medicine, psychiatry	2
9E	Executive Leader	COS, DEO, ACOS/E	3
9 T	HPT	Medical student, PGY-2, PGY-3	3
10C	Chief	Medicine, psychiatry 1, psychiatry 2, surgery	4
10E	Executive Leader	COS, DEO	2
10 T	HPT	PGY-1 (2), PGY-3, fellow (2)	5
10T_s**	HPT	Medical student	1

*COS*, Chief of Staff; *DEO*, designated education officer; *HPT*, health professions trainee

*Role is defined for HPTs by educational level, for educational site leadership by position, and for service chiefs by discipline

**Denots an Interview with an individual participant (*s*, medical student; *r*, resident; *f*, fellow)

### Procedures

Three evaluation team members with qualitative experience (SMN, LMK, NWB) conducted semi-structured interviews via Microsoft Teams videoconferencing software between May and August 2021. Three parallel interview guides were created for each interview group (Supplemental Digital Content) in collaboration with national VA HPT program leadership. Interview questions asked about the ways that HPTs contributed to VA’s patient care efforts and the role of HPTs in their site’s response to COVID-19. All interviews were digitally recorded and lasted between 23 and 76 min. Audio files were transcribed using a VA-approved transcription service and de-identified.

### Analysis

Data was analyzed iteratively using descriptive content analysis^21,22^. Analysis involved a combination of deductive coding with codes derived from the interview guide and inductive coding with codes that emerged from the data.^23^ First, four evaluation team members (SMN, GMY, JSD, LMK) read through four transcripts to identify an initial set of codes. Saturation was reached when codes became redundant, and no new codes were observed. From there, the team split into pairs, with each coding a portion of the dataset, and each member working independently. Upon completion of their coding, the pair met to discuss discrepancies and to modify or expand the codebook when necessary. When the team could not meet consensus, they consulted a third team member. After the team coded the data, the senior author (LMK) reviewed coding reports to identify meaningful domains and subsequently discussed with the analysis team, where final domains were revised based on comments. Transcripts were coded using NVivo 12.0 software.^24^

### Ethical Concerns

This evaluation was designated as non-research quality improvement study in accordance with VA guidance, Office of Research and Development (ORD) Program Guide 1200.21^25^ and was therefore exempt from IRB oversight. However, steps were taken to ensure that the safety and welfare of interview participants were appropriately protected, including obtaining verbal consent to record from participants prior to the start of each interview and the use of a secure VA server to protect all data. Findings were deidentified to protect confidentiality.

## RESULTS

Two domains were identified from participant responses: the influence of COVID-19 on HPT programs and the contributions of HPT trainees to VA COVID-19 response, with themes presented below accordingly.

### Influence of COVID-19 on HPT Programs

#### Decreased Learning Opportunities and Safety Concerns

Participants discussed COVID-19 pandemic-related restrictions on training opportunities as differentially affecting HPTs based on their years of training. All participant groups expressed a common sentiment that medical students, the most junior trainee group, had little opportunity to be involved in patient care activities. Restrictions influenced medical students, residents, and fellows in multiple ways, including being rapidly pulled from their VA sites by their affiliates, reduced patient care opportunities, reduced interactions with attending physicians, and limited opportunities for involvement in learning and research. As two service chiefs described:

The Association of American Medical Colleges made a decision fairly early on to pull medical students out. I happen to think that that was a very ill-advised decision. All of you can listen when all of the post hoc analysis comes out. I think I will be shown to be correct. I’m not saying that medical students could have cared for COVID patients, but I think they could have done a lot and learned a lot. And I think pulling them away from what is an absolutely unprecedented clinical experience in our world was a big mistake. (Medicine service chief)

What really negatively influenced them a lot is the decline of the number of operative cases for them. Which I think is going to have a relatively long-term consequences for this class. They spend several months without the volume of cases they needed, especially they’re graduating and they’re not going to have time to make up for this, it was just kind of an interesting thing. And in fact, the ACGME lowered down the requirements for graduation as far as the operative cases because of this. (Surgery service chief)

Medical student HPTs also provided their perspective on the restriction of training opportunities:

I have not treated a COVID patient since the beginning of the pandemic. Towards the beginning, med students were definitely shielded away from them. And now, just because of the ambiguity of whether or not I’m allowed to see COVID patients, the supervisors on my team have just been like, ‘Oh, this is a COVID patient? Medical Student, do not see them.’ (Medical student)

I was here in, I guess, March or February before there were any cases of COVID here. We were removed, not by any doing of the VA but because of the medical school. I didn’t get to go in and help with any patients then. Then, when I was here again in August afterwards, we just simply didn’t have any COVID patients, I can’t really comment on anything about the medical students’ role in that. We haven’t had any, now that I’ve been here, in the spring, summer, either. (Medical student)

In addition to reduced opportunities for training, participants discussed how safety considerations and changes in workflow protocols influenced HPT-delivered clinical care:

The past year has been a little bit challenging. First with the pandemic, they pulled the trainees out, or the majority of academic affiliates did. And then some of the academic affiliates had them come back to some capacity, some did not. So, that was kind of in this constant state of flux. But that wasn’t a barrier that any of us foresaw, and obviously we had to adapt. But it does pose other questions in terms of just education of trainees, and safety, and do we send them into COVID positive rooms? Do we not? Are we making sure that we’re properly trained with the safety equipment? I think we have a lot more emphasis on that now than we did prior to the pandemic. But the pandemic put a whole weird spin on interaction with trainees, that just was not anticipated at all. (Medicine service chief)

### Contributions of HPTs to VA COVID-19 Response

#### HPTs Provided Hands-on Clinical Care

While less advanced HPTs faced restrictions on their ability to engage in clinical care, more advanced HPTs were heavily involved with VA in-person care delivery efforts related to COVID-19 and non-COVID-19 medical services, as discussed by multiple participants. Two advanced HPTs mentioned HPT assistance with COVID-19 related care:

Especially on the Intensive Care level of care, any of the response to COVID was 100% reliant on the trainees flexing up the numbers of patients that they were taking care of in the ICU [Intensive Care Unit]. (Fellow trainee)

It was maybe overwhelming emotionally, just because I still remember, by that time, I had had maybe two or three patients pass away on my teams, and it was the first time – the first week of when I started having COVID patients, it was like three deaths in a week, and it was like, “Well, I guess this is what’s happening.” But from a medicine perspective, really, we just started seeing more patients with hypoxic respiratory failure, but nothing else changed. It was the volume and the severity of the patients, but you kinda see patients as sick as that in the ICUs. (PGY-3 trainee)

Site leadership also acknowledged the contribution of HPTs with non-COVID-19 care:

They helped with the patient volume so the attendings could see the face-to-face care of the COVID patients. We did separate them off, in which just from an infection control perspective throughout – so, the COVID patients were separated off. They did perform some virtual care. But they did perform a lot of the non-COVID care that enabled the COVID-specific attendings to care. (DEO)

HPTs also positively contributed to VA patient care efforts by alleviating some of the workload of VA attending physicians. For example, a service chief recalled how, during the early days of the pandemic, two of the site’s Chief Residents stepped up to take on additional work, which helped with attendings’ workload:

I’m speaking mostly of that period March to June of 2020 when we were hit the hardest, I don’t know if we could have done it without our two chief residents. They were both credentialed and privileged, so they actually stepped up to be hospitalist attendings during that time, and that allowed me to move to a 7 on, 7 off model for my hospitalists, which allowed me to…It just allowed me to switch to a schedule that allowed me to rest my staff and give people a way to kind of have a break during a very stressful time. And they also organized the residents*.* (Medicine service chief)

#### HPTs Supported VA’s Shift to Telehealth

Another way in which HPTs supported VA’s pandemic response was by contributing to VA’s shift to telehealth. Several service chiefs and educational leaders mentioned that HPTs supported VA during the COVID pandemic by providing virtual care.

For psychiatry, we transitioned relatively rapidly to mostly virtual care. Aside from people who presented to the ER in a state of crisis or emergency and required acute admission, we trained the residents on televideo or telephone, and they pretty much carried on with virtual care. (Psychiatry service chief)

I think the biggest thing was for them to experience the remote virtual care modality, especially for trainees. They’re not used to this. It’s all face-to-face. That gave them the opportunity to experience this where they could through talking to patients on the phone or through video, be able to make some assessment and some decisions along with their supervisor attending. (Chief of Staff)

HPTs were also called out as contributing to the VA health care system’s overall shift toward offering more telehealth services.

I can tell you that before the pandemic, we hardly ever did VVCs [VA Video Connect] for telemedicine. We did few, but not a lot. This one really opened up the doors, and now, it’s becoming part of the routine. (Physical Medicine and Rehabilitation service chief)

Another notable finding pertaining to this shift to telehealth was that a couple educational leaders mentioned trainees ability to easily adapt to new technology as well as share their knowledge about technology and problem-solve to identify solutions to barriers to VA’s rapid virtual care adoption. As one DEO shared:

The trainees were really a great inspiration to us. I mean, they quickly adapted to things like virtual healthcare, and we continued to deliver virtual health in both our primary care and specialty clinics. They were instrumental in helping us think about, particularly with video visits, how to creatively do physical exams, collect data…ensure that patients were getting taken care of in a new way. (DEO)

#### HPTs Provided Informational and Emotional Support

Another way in which HPTs supported VA’s COVID-19 response efforts was through HPTs’ informational and emotional support to Veteran patients. This support included things like providing medical updates and bridging communication with families who were not allowed to visit in person. Such support was particularly influential early in the pandemic when opportunities to engage in patient care were limited and other VA provider groups were over-extended based on increased patient care demands. A few participants described how HPTs made contributions to VA’s COVID-19 response using support activities such as phone calls with patients and families:

Another big thing, too, is calling the families and explaining … what was going on. There’s a lot of decision making that was had remotely. There was also a lot of communication. Early on, VA’s response, and not just the VA, many hospitals, because family members weren’t allowed in. While some of these people were expiring, you would have to handle that over the phone. That was a big part. The trainees responded, and it brought a little humanity to the pandemic. (PGY-1 trainee)

I think the other thing that they really should get kudos for because again, I said, our trainees are our frontline providers and I saw that they stayed late to inform Veterans’ families on the progress when families couldn’t come in. They would spend the time connecting them through FaceTime or through other applications to ensure that the connections. (DEO)

When we were limited to urgent, emergent, and time-sensitive cases, they [trainees] did mind all the phones with us in order to help us reschedule cases, talk to patients, evaluate the patients just to make sure that the conditions hadn’t changed, and so on. (Surgery service chief)

#### HPTs Contributed to a Positive Work Environment

HPTs were also noted as making important contributions to a positive work environment when VA employees were stressed about COVID-19. Discussed by a few site leaders and service chiefs, HPTs were viewed as having been flexible, collegial, and a boost to employee morale.

The other thing that I think the trainees did is they connected inter-professionally so leaning on each other with nurses and respiratory therapists was actually, it just made for a very collegial environment when we couldn’t control the stressors. (DEO)

I think the trainees were really a great inspiration to us. (Psychiatry service chief).

Everything went smooth, and they were very receptive, flexible. They were great help. (Psychiatry service chief)

Notably, HPTs were described as helping to create a positive work environment not only during COVID, but also during times of stability.

#### HPTs Contributed to COVID Planning and Decision-making

Two participants also noted that HPTs contributed to VA’s COVID response planning efforts, assisting with developing protocols around caring for COVID patients and protecting other HPTs. This contribution was viewed favorably by the participants, who were not HPTs, and was considered particularly important given HPTs’ role in providing overnight coverage.

And honestly, it was our chief resident of quality and patient safety that I really believe provided outstanding guidance during our COVID response with respect to safety issues, not just for patients but for our residents. They developed very detailed protocols around caring for COVID patients outside the ICU. We were 100 percent COVID at the initial surge. Redeployment, I mean, that type of innovation, it was wonderful to watch. (DEO)

The residents were involved in helping us with how to protect residents, how to keep people safe and going through all our respiratory precaution protocols. I think they were – Necessarily we involved them in planning because, as [Medicine service chief] said, a lot of time they’re here more at night than we are. They need to be protected. (Surgery service chief)

## DISCUSSION

This evaluation provides the only known qualitative account of HPT and training leadership experiences within VA training placements during the COVID-19 pandemic that includes multiple participant groups. Given VA’s national scope and its status as one of the largest training placements in the USA, these findings offer a rare and focused look at how large learning healthcare systems navigated trainee roles during a public health crisis. This study offers unique insight into HPT contributions in emergency response settings—particularly around technological adaptation and support for staff morale—areas that have been largely underexplored in existing literature. Rather than highlighting only the disruptions to training, as prior research often does,^6,8,15,17,27–31^ this evaluation shows how HPTs meaningfully bolstered the VA’s ability to meet the challenges of the pandemic. Their roles extended beyond direct patient care to include rapid technological adaptation, emotional support to patients, a boost to staff morale, and input on pandemic planning and decision-making—revealing a multi-faceted and underappreciated benefit of trainee involvement during crisis response.

One notable and novel finding from this evaluation is the emotional support HPTs provided to both patients and clinical staff. The topic of HPT contribution to employee morale is also not present in any existing literature focused on emergency response. Literature more often highlights disruptions to training^6,8,15,16,26–30^ and how COVID-19 heightened feelings of isolation among trainees^31^ rather than trainees’ own contributions to the clinical care system. Participants described how HPTs’ presence helped lessen the emotional toll of the pandemic by offering reassurance to patients and fostering morale among overburdened healthcare teams. It is possible that these contributions may have stemmed from strong relationships with faculty and the shared increased sense of purpose in serving during a public health emergency. It is also possible that the lesser leadership burden on trainees allowed for them to better assume emotionally supportive roles. Recognizing this role opens new avenues for better integrating HPTs into future crisis planning—not only as learners but as stabilizing forces in the healthcare system.

In addition to contributions to morale and emotional support, HPTs also played a key role in supporting the rapid shift to telehealth. Participants emphasized that trainees’ comfort with digital tools and creative problem-solving directly facilitated the transition to virtual care. This finding stands out given the limited literature on HPTs’ role in technological adaptation during crises and is especially important considering that many trainees were moved from in-person care to telehealth due to PPE shortages.^30,32^ These insights suggest that crisis response policies could more deliberately leverage HPTs’ technological ability to accelerate the implementation of virtual or technological care.

Also previously unexplored in existing literature was the role of trainees in planning pandemic response efforts. Residents assisted with developing protocols around caring for COVID patients outside the ICU and plans to protect other residents. As healthcare leaders reflect on previous pandemic preparations that have been helpful along with opportunities for improvement, opportunity exists to involve trainee representatives in the development of emergency or disaster response preparations.

Taken together, these findings underscore the need for emergency response frameworks to account for the diverse strengths of trainees. Future efforts might include formal training for HPTs in crisis communication and emotional support, as well as roles specifically designed to enhance morale and technological agility in times of disruption. Integrating these functions into emergency preparedness could amplify both patient care and workforce resilience.

This evaluation had limitations, though also highlights opportunities for additional study. One limitation of the present study is that data collection focused on personal perspectives and did not include a review of concrete markers of influence, such as research or clinical productivity. While the lack of objective measures is a drawback, this qualitative study provides rich insight into the trainees’ role in COVID-19 response, particularly in areas less typically documented such as morale and technological adaptation. Future research should assess concrete markers of HPT influence on COVID-19 response (e.g., number of care hours provided by HPTs) as well as causal relationships between measures of interest.

Additionally, this evaluation was not designed to assess the extent to which participant perspectives represent other groups in VA. The present study focused on one group of medical student and physician HPTs and may not reflect the experiences and contributions of HPTs from other clinical disciplines. Future research could also benefit from including additional groups, such as trainees in mental health settings and clinical educators, to explore HPT contributions from additional viewpoints. It may be useful to explore concerns and limitations experienced on the affiliate side, which may provide insight into other factors contributing to decreased training opportunities, including perceived lack of transparency or shared decision-making. Such work, supplemented by findings presented here, could be used to inform VA and affiliate policies regarding handling HPT clinical care involvement during sudden increased care needs.

An additional limitation is that the group interview guide was not designed to elicit detailed descriptions of the mechanisms through which HPTs exerted influence or the degree to which HPTs exerted influence, which limited the ability to fully explore specific contributions and barriers across several key domains. For example, while participants emphasized the importance of HPTs’ contributions to morale and interprofessional collaboration, the data lacked sufficient detail to identify specific strategies or behaviors driving these outcomes. Similarly, participants noted that HPTs supported the transition to telemedicine through their technological knowledge and adaptability but did not consistently elaborate on how that support was operationalized, such as whether HPTs engaged in direct instruction of faculty or staff regarding the video platforms, or why they believed HPTs to be more technologically proficient. It is possible that HPTs’ perceived comfort with technology was due to their educational background; however, this warrants further investigation. It was also beyond the scope of this evaluation to determine whether decisions were made from the top-down or in partnership with some trainees, and the degree to which HPTs had input into decision making that impacted their clinical placements or training opportunities. Future research should evaluate whether greater shared decision-making might mitigate negative training impacts in future times of crisis. Notably, follow up focus groups with HPTs could delve more into the underpinning mechanisms within each of these topics, investigating specific suggestions to mitigate the reduction in training opportunities in the event of emergency crises, reasons why HPTs were perceived as particularly technologically proficient, barriers and facilitators to HPTs sharing their technological expertise, and what shared decision-making might look like in planning for future emergency responses.

In addition to the areas noted above, opportunity exists for further investigation into how trainees contribute to employee engagement in times of crisis. Future research could evaluate how training programs can better leverage HPT contributions to boost staff morale and provide technical expertise in crisis situations, including by evaluating specific ways in which HPTs contribute to these domains. Future research could also assess how HPTs participate in clinic-level technological growth, and whether institutional structures (e.g., peer teaching opportunities, informatics rotations) can support and even expand these roles. Additional work could explore how to provide training and structure to enable HPTs to more fully contribute to these areas.

## CONCLUSIONS

This evaluation highlights the crucial and often overlooked role of HPTs in supporting emergency response efforts within a large, complex healthcare system. Within the context of the COVID-19 pandemic, HPTs played a significant part in facilitating the rapid move to telehealth technologies, providing emotional support to Veterans, and enhancing staff morale during a period of unprecedented stress.^26^ These findings underscore the adaptability of HPTs, their capacity for assistance in creative problem-solving, and their potential to meaningfully contribute to healthcare delivery in crisis situations. While situated within the VA context, the insights from this evaluation have broader relevance for healthcare systems preparing for future emergencies. Rather than limiting trainee involvement during crises, organizations should consider structured, intentional strategies to engage HPTs in ways that leverage their adaptability, technical fluency, and capacity to support both patients and providers. As emergency response planning evolves, recognizing and utilizing the full scope of HPT contributions can enhance healthcare system responsiveness, improve patient outcomes, and support workforce well-being during times of rapid change.

Taken together, these findings suggest a need for a policy framework that proactively defines and supports the role of HPTs during times of crisis. Beyond simply allowing trainees to remain involved, healthcare systems should establish protocols and infrastructure that enable HPTs to contribute meaningfully to emergency response—such as through structured roles in telemedicine innovation, morale-building initiatives, disaster preparedness planning, or technology integration efforts. Embedding HPTs within these efforts can expand the system’s capacity while simultaneously enhancing the training experience. Policymakers, clinical educators, and health system leaders should consider how to systematically integrate HPTs into crisis planning to ensure their contributions are not only permitted but leveraged in strategic, sustained ways.

## Supplementary Information

Below is the link to the electronic supplementary material.

ESM 1 (DOCX 22.8 KB)

## Data Availability

Data are not appropriate for public-use sharing.

## Declarations

None. No outside data were used in this study.
