# Supplementary material for: COVID-19 and VA Medical Education Training Programs: Influence on Trainees and Contributions of Trainees to VA’s Pandemic Response
Source: J Gen Intern Med. 2026 May 27;41(14):4012–20. doi: 10.1007/s11606-026-10522-3 (PMC13728052; doi:10.1007/s11606-026-10522-3)
Supplement: Supplementary file 1 [file 11606_2026_10522_MOESM1_ESM.docx]

**Supplemental Content. Participant Interview Guides.**

Hello! This is [name] from the QUERI Center for Evaluation and Implementation Resources, otherwise known as CEIR. Thank you for taking the time to speak with me/us today!

*[Introduce yourself; invite second interviewer to also introduce themself]*

The purpose of today’s interview is to help us understand your perspectives about the Office of Academic Affiliations’ training programs for medical students and residents. CEIR is conducting a *rapid impact evaluation* of OAA’s training and education programs, focusing on the impact of these programs on VHA trainees and supervisors, as well as upon the broader VHA system of care. During our conversation, we will cover topics such as the ways in which trainees impact the work experiences of VHA staff at your site, the contributions that trainees at your site make to research, innovation efforts, and patient care, and the ways that your site promotes VHA as a place for a post-training career. This interview has been designed to take no more than one hour of your time today.

It is important to clarify that, per VHA Guidance 1200.21, this interview is considered non-research. Evaluation findings will be used to develop a report provided to OAA; findings will also serve to support additional evaluation work on the impact of OAA’s education and training programs on the VHA system of care.

Your participation in this interview is completely voluntary. If at any time you would prefer not to answer a question, please feel free to decline to answer. Also, if at any time you would prefer to no longer participate, any responses you did give will not be included in the evaluation. Please be assured that any information that you share today will be kept confidential and aggregated in any products that result from this evaluation; you will not be identified in any reporting.

We do value your time and feedback and would like to record the interview so that we do not miss anything that you say.

Do we have your permission to record this interview?

*[Wait for participants to confirm]*

We ask that you please refrain from mentioning individual names; however, if any are brought up, they will be removed from reporting to further maintain privacy and confidentiality. Please do not include any patient specific information during the interview.

To allow our conversation to flow more freely and to assist with transcription, we ask the following:

1. Only one person should speak at a time. If you have something important to say and we have not called on you, you can get our attention by clicking the small smiley face with a raised hand at the top of your screen.
2. Everyone does not have to answer every single question; however, we would like to hear from each of you today as the discussion progresses.
3. We ask that you please refrain from sharing any information provided by other members of your interview group.
4. If there is something you are not comfortable saying in front of the group, you are welcome to share comments with us privately after the call. Just send one of the interviewers an email.
5. Please keep in mind that non-verbal communication, such as nodding to indicate affirmation, cannot be transcribed.
6. There are no “right or wrong answers,” just different opinions. Please say what is true for you, even if you are the only one who feels that way.

Do you have any questions before we begin the interview?

*[Answer questions.]*

In a moment, I will start recording. You will hear me say my name, the date, and your group’s evaluation ID number.

*[If the participant agrees to participate, start the recording, state your name, the date, and the evaluation ID number, and begin the interview.]*

1. **Opening**

- (trainee) To begin, please tell me, briefly, what your roles are at your VHA medical center.
- (educational leadership) To begin, please tell me, briefly, what your roles are at your VHA medical center.
- (clinical service chiefs) To begin, please tell me, briefly, what your roles are at your VHA medical center.

2. **Retention & Recruitment**

- (trainee) What things does your site do to promote VHA as a place for a post-training career?
  - Follow-up: What role have your interactions with mentors or supervisors played in your view of VHA as a place for a post-training career?
  - Follow-up: In what ways can your site improve in its promotion of VHA as a place for a post-training career?
- (educational leadership) What things does your site do to promote VHA as a place for a career for its trainees?
  - Follow-up: Have your training programs enhanced recruitment efforts at [site]? If so, how have they enhanced recruitment efforts?
  - Follow-up: What impact, if any, have your training programs had on retention of current staff at your site?
  - Follow-up: What best practices are either in place or missing at your site to support recruitment and retention?
- (clinical service chiefs) What things does your department do to promote VHA as a place for a career for its trainees?
  - Follow-up: Has your training program enhanced recruitment efforts in your department? If so, how has it enhanced recruitment?
  - Follow-up: What impact, if any, has your training program had on retention of current staff in your department?
  - Follow-up: What best practices are either in place or missing in your department to support staff recruitment and retention?

3. **Research, Innovation, and Other Non-Clinical Activities**

- (trainee) What contributions have trainees at your site made to research, innovation, and other non-clinical activities, such as quality improvement and program management?
  - What barriers exist to involving trainees in these efforts?
  - In what ways could your site improve in this area?
- (educational leadership) In what ways have trainees at your site offered meaningful contributions to research, innovation, and other non-clinical activities?
  - What barriers exist to involving trainees in these efforts?
  - In what ways could your site improve in this area?
- (clinical service chiefs) In what ways have trainees in your department offered meaningful contributions to research, innovation, and other non-clinical activities?
  - What barriers exist to involving trainees in these efforts?
  - In what ways could your department improve in this area?

4. **Patient Care**

- (trainee) In what ways do trainees impact patient care at your site?
  - Follow-up: In what ways do trainees impact the workload at [site]?
  - What barriers exist to involving trainees in patient care?
  - In what ways could your site improve in this area?
- (educational leadership) In what ways do trainees impact patient care at your site?
  - Follow-up: In what ways do trainees impact the workload at [site]?
  - What barriers exist to involving trainees in patient care?
  - In what ways could your site improve in this area?
- (clinical service chiefs) In what ways do trainees impact patient care at your site?
  - Follow-up: In what ways do trainees impact the workload at [site]?
  - What barriers exist to involving trainees in patient care?
  - In what ways could your site improve in this area?

5. **Work Culture**

- (trainee) We have already asked you about how your work impacts patients. Now we would like to hear how your work impacts other VA employees. How does the work you do as a trainee impact the experience of others working at your site?
  - Follow-up: What could your site do to assist you in this area?
- (educational leadership) In what ways have *trainees* impacted the experience of others working at your site?
  - Follow-up: In what ways do your *training programs* impact the experience of others working at your site?
- (clinical service chiefs) In what ways have *trainees* impacted the experience of others working at your site?
  - Follow-up: In what ways does your *training program* impact the experience of others working at your site?

6. **Support Staff**

- (trainee) What type of administrative support staff is needed at your site, but not currently present, to support your training program?
  - What things could trainees at your site do if they had more administrative support staff?
- (educational leadership) What type of support staff is needed, but not currently present, to support your training programs?
  - What things could your site do if you had more support staff?
- (clinical service chiefs) What type of support staff is needed, but not currently present, to support your training program?
  - What things could your program do if you had more support staff?

7. **COVID-19 and Training Impact**

- (trainee) How has the work of trainees been a part of your site's response to COVID-19?
- (educational leadership) What role have trainees played in your site’s response to COVID-19?
- (clinical service chiefs) What role have trainees played in your site’s response to COVID-19?

8. **Closing**

- (trainee) Given our interest in evaluating OAA’s impact on the VHA system of care, what else would you like to share about your training experience?
- (educational leadership) Given our interest in evaluating OAA’s impact on the VHA system of care, what else would you like to share about your site’s training program?
- (clinical service chiefs) Given our interest in evaluating OAA’s impact on the VHA system of care, what else would you like to share about you/your department’s training program?
